# Clinical Implications of Co-Inhibitory Molecule Expression in the Tumor Microenvironment for DC Vaccination: A Game of Stop and Go

**DOI:** 10.3389/fimmu.2013.00417

**Published:** 2013-12-03

**Authors:** Angela Vasaturo, Stefania Di Blasio, Deborah G. A. Peeters, Coco C. H. de Koning, Jolanda M. de Vries, Carl G. Figdor, Stanleyson V. Hato

**Affiliations:** ^1^Department of Tumor Immunology, Nijmegen Centre for Molecular Life Sciences, Radboud University Nijmegen Medical Centre, Nijmegen, Netherlands; ^2^Department of Medical Oncology, Nijmegen Centre for Molecular Life Sciences, Radboud University Nijmegen Medical Centre, Nijmegen, Netherlands

**Keywords:** DC vaccination, tumor microenvironment, checkpoint blockade, tumor-specific T cells, cancer treatment

## Abstract

The aim of therapeutic dendritic cell (DC) vaccines in cancer immunotherapy is to activate cytotoxic T cells to recognize and attack the tumor. T cell activation requires the interaction of the T cell receptor with a cognate major-histocompatibility complex-peptide complex. Although initiated by antigen engagement, it is the complex balance between co-stimulatory and co-inhibitory signals on DCs that results in T cell activation or tolerance. Even when already activated, tumor-specific T cells can be neutralized by the expression of co-inhibitory molecules on tumor cells. These and other immunosuppressive cues in the tumor microenvironment are major factors currently hampering the application of DC vaccination. In this review, we discuss recent data regarding the essential and complex role of co-inhibitory molecules in regulating the immune response within the tumor microenvironment. In particular, possible therapeutic intervention strategies aimed at reversing or neutralizing suppressive networks within the tumor microenvironment will be emphasized. Importantly, blocking co-inhibitory molecule signaling, often referred to as immune checkpoint blockade, does not necessarily lead to an effective activation of tumor-specific T cells. Therefore, combination of checkpoint blockade with other immune potentiating therapeutic strategies, such as DC vaccination, might serve as a synergistic combination, capable of reversing effector T cells immunosuppression while at the same time increasing the efficacy of T cell-mediated immunotherapies. This will ultimately result in long-term anti-tumor immunity.

## Introduction

The goal of cancer immunotherapy is to activate, or reactivate, the immune system in cancer patients for therapeutic benefit. This is a challenging endeavor, as escape from immunosurveillance is an essential requirement for tumor progression. Early tumors can be eliminated or contained by the immune system but, by a process involving immunoediting, tumor cells can eventually escape this detection (1). They do so by hiding from immune detection, blocking the function of immune cells, and/or by influencing immune cells to induce tolerance to the tumor and even to produce tumor growth enhancing factors. Despite this escape from immunosurveillance, there is ample evidence indicating that it is possible to induce specific anti-tumor immune responses either naturally (spontaneous) or therapeutically. This requires a number of discrete steps. Firstly, dendritic cells (DCs) must take up and present antigens derived from the tumor, which can be encountered *in situ* or delivered to the DCs *ex vivo* as part of a therapeutic vaccine. This has to be coupled to an activation or maturation signal to the DC. Next, these mature tumor antigen presenting DCs migrate toward the lymphoid organs, where they have to induce antigen-specific T cell responses that target the tumor (2, 3). Efficient anti-tumor responses are believed to require CD8^+^ cytotoxic (killer) T cells, but recent data indicate that induction of CD4^+^ T helper cells also contribute to clinical efficacy (4). Conversely, DCs may also trigger antibody and natural killer (NK) cell responses, which can contribute to anti-tumor immunity (5, 6).

**Figure 1:**
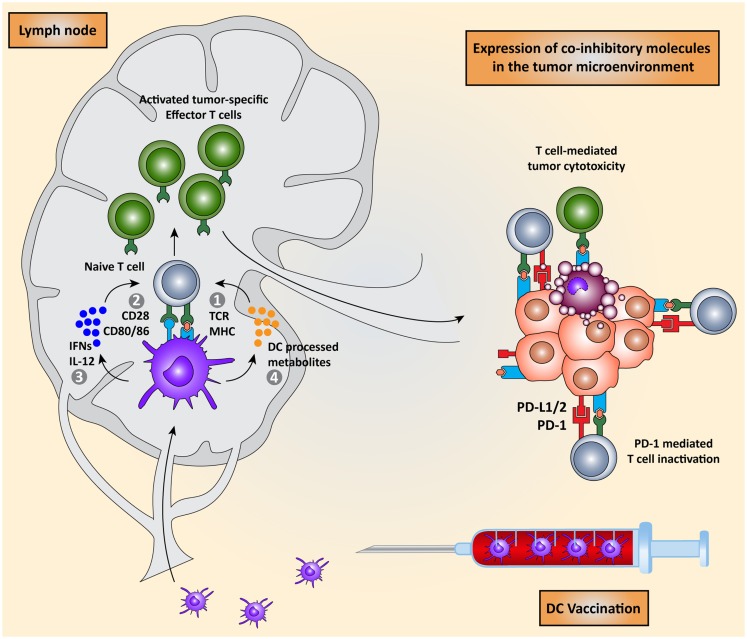
**Dendritic cell vaccination is counteracted by host immunosuppressive mechanisms**. Monocytes or natural occurring dendritic cells are isolated from the peripheral blood of the patient, loaded with tumor antigens, and subsequently matured. These activated DCs are re-infused into the patient and migrate to the lymph node to encounter and interact with naïve T cells in order to induce the activation of effector T cells. DC-mediated T cell activation is regulated by four signals: (I) interaction between TCR on T cells and MHC:peptide complex, (II) co-stimulation via CD28 and CD80/86 expressed on T cells and DCs respectively, (III) secretion of pro-inflammatory cytokines such as IFNs and IL-12, and (IV) release of DC-processed metabolites. These activated CD8^+^ cytotoxic T cells and CD4^+^ T helper cells migrate to the tumor site where they are eventually neutralized by the immunosuppressive nature of the tumor microenvironment due, for instance, to the expression of co-inhibitory molecules.

Priming of naïve T cells into antigen-specific effector T cells by DCs requires four signals (Figure 1): (I) engagement of a T cell receptor (TCR) with a peptide-major-histocompatibility complex (MHC) on the DC and (II) the right balance between expression of co-stimulatory molecules that activate T cell proliferation and co-inhibitory molecules that attenuate T cell activation on both cell types. (III) A third signal is provided by cytokines secreted by the DCs, which promote T cell differentiation and polarization toward specific effector T cell phenotypes. Finally (IV), DCs regulate the induction of specific chemokine receptors and integrins on T cells to direct migration toward specific tissues (2, 7–10).

The above-described induction of T cell-mediated anti-tumor immunity can be exploited therapeutically in several ways, the two most popular being DC vaccination strategies and adoptive T cell transfer. These intervention strategies are referred to as cell-based immunotherapy and both rely on the isolation of autologous immune cells from a patient followed by *ex vivo* manipulation and then re-infusion into the patient. In recent years, much progress has been made in this field: tumor antigens, DCs, and T cells, as well as adjuvants have been optimized, leading to an increase in the number of patients with an anti-vaccine immune response. However, despite these improvements, the clinical responses are still limited. This is most likely caused by the establishment of an immunosuppressive tumor microenvironment. As such, to further improve immunotherapeutic approaches, strategies to neutralize immunosuppression are required. A promising strategy, and the main subject of this review, involves the manipulation of co-stimulatory and co-inhibitory molecules to change the balance within the tumor microenvironment from an immunosuppressive state into an immunostimulatory state.

We will first discuss the current state of DC vaccination, followed by how these therapies could be affected by the immunosuppressive tumor microenvironment. Subsequently, we will review current strategies for reversing the immunosuppressive state of the tumor microenvironment, which are in clinical or pre-clinical stage. We will conclude by discussing the merits of combining DC vaccination with blockade of immune checkpoints in cancer treatment.

## Dendritic Cell Vaccines

Dendritic cells are the most potent antigen presenting cells (APCs) and provide a key functional link between innate and adoptive immune responses. In their immature state, they take up and process antigens in the peripheral blood and tissue, then undergo maturation and migrate to lymphoid organs where they present the antigens to naïve T cells (11). These mature DCs, now expressing high levels of cell surface MHC class I and II molecules, can activate both naïve CD8^+^ cytotoxic T cells and naïve CD4^+^ T helper cells (12–14) in a process dependent on the upregulation of co-stimulatory molecules such as CD40, CD80, CD86, and OX40L on the APC surface (7, 15). These molecules interact with corresponding ligands expressed on T cells (Figure 1), with the interaction between CD86 on DCs and CD28 on T cells being the most significant to trigger T cell activation and expansion (16, 17). Conversely, T cells and DCs also express co-inhibitory molecules, such as the receptors programed cell death-1 (PD-1) and the cytotoxic T lymphocyte-associated antigen-4 (CTLA-4) expressed on T cells and the ligands PD-ligand 1 (PD-L1) and PD-ligand 2 (PD-L2) present on DCs. The interaction between these co-inhibitory molecules can inhibit T cell priming and activation and the delicate balance between co-stimulation and co-inhibition determines the fate of a T cell response. The expression and regulation of these proteins on DCs and T cells have been recently reviewed (2). During the process of co-stimulation, DCs secrete cytokines that regulate the differentiation of naïve T cells into different subsets of effector T cells, in particular CD4^+^ T helper cells. This process results in the differentiation toward a Th1, Th2, Th9, Th17, or regulatory T cell (Treg) phenotype (18). Lastly, environmental cues from the DCs, such as DC-processed metabolites, provide T cells with a signal to home, and migrate to certain tissues (19).

Therapeutic DC vaccination strategies against cancer aim to exploit the ability of DCs to prime antigen-specific T cells, in order to induce a T cell-mediated, specific, immune response that targets and destroys the tumor. DCs, for example naturally occurring blood DCs or *ex vivo* generated monocyte-derived DCs, are provided with tumor-specific antigens, either by loading them *ex vivo* with the tumor peptides and then injecting the cells back into the patient or by targeting them *in vivo* (3, 20–22). At first, DC vaccination protocols mainly focused on targeting cytotoxic CD8^+^ T cells, but it has become clear that CD4^+^ T cells not only augment the induction and proliferation of these CD8^+^ T cells, but also participate in the elimination of tumor cells and maintenance of long-term immunity. Thus an efficient vaccine should be able to induce both CD8^+^ and CD4^+^ T cells. Vaccination with MHC class I/II-loaded DCs has been shown to both increase the frequency of tumor-specific CD8^+^ T cells and co-activate CD4^+^ T cells, thereby further improving clinical responses (4, 23).

Recently, the first commercial DC vaccine, Sipuleucel-T, was approved by the FDA for the treatment of prostate cancer. In a phase III clinical trial, Sipuleucel-T showed an increase of 4.3 months in median survival and 33% reduction in the risk of death (24). Nevertheless, despite the significant benefit in median survival, satisfying clinical effects in terms of solid anti-tumor immune responses were only observed in a minority of patients, strongly suggesting that further optimization is warranted (25). Other trials also underscore the potential of DC vaccination in metastatic cancers, especially in melanoma. In this setting, it was shown that autologous DCs loaded with tumor antigens are safe and capable of inducing tumor antigen-specific immune responses in a substantial part of the vaccinated patients (26). Despite these growing successes, DC vaccination has not yet proven to be a method superior to other protective immunity stimulating vaccine strategies (27, 28). Anti-tumor responses are hampered by the tumor microenvironment which seems to be very immunosuppressive, especially in patients with a high tumor load (20, 29).

## Immunosuppressive Tumor Microenvironment

Although DC vaccination succeeds in activating the immune system, resulting in the presence of tumor-specific T cells, the clinical success of these treatments is still limited. The lack of clinical efficacy can be mostly attributed to an immunosuppressive tumor microenvironment, which is very successful in attenuating T cell-mediated responses. The tumor microenvironment consists of tumor cells, fibroblasts, endothelial cells, and infiltrating immune cells together with extracellular matrix components. Infiltrating immune cells can be either beneficial or detrimental depending on the nature of the infiltrating cells. The presence of tumor-infiltrating lymphocytes (TILs) has been associated with improved survival of patients with prostate, breast, colorectal, ovarian cancer, or melanoma (30–33). On the other hand, the presence of Tregs or myeloid-derived suppressor cells (MDSCs), which can inhibit anti-tumor immune responses, is associated with decreased survival (34–36). Furthermore, tumor cells express a number of proteins on their cell surface capable of inactivating tumor-specific T cells, as detailed below. Therefore, immunotherapy strategies aimed at inducing T cell-mediated anti-tumor immunity need to include an approach to break tolerance to the tumor.

### Inhibitory checkpoint receptors and ligands

**Figure 2:**
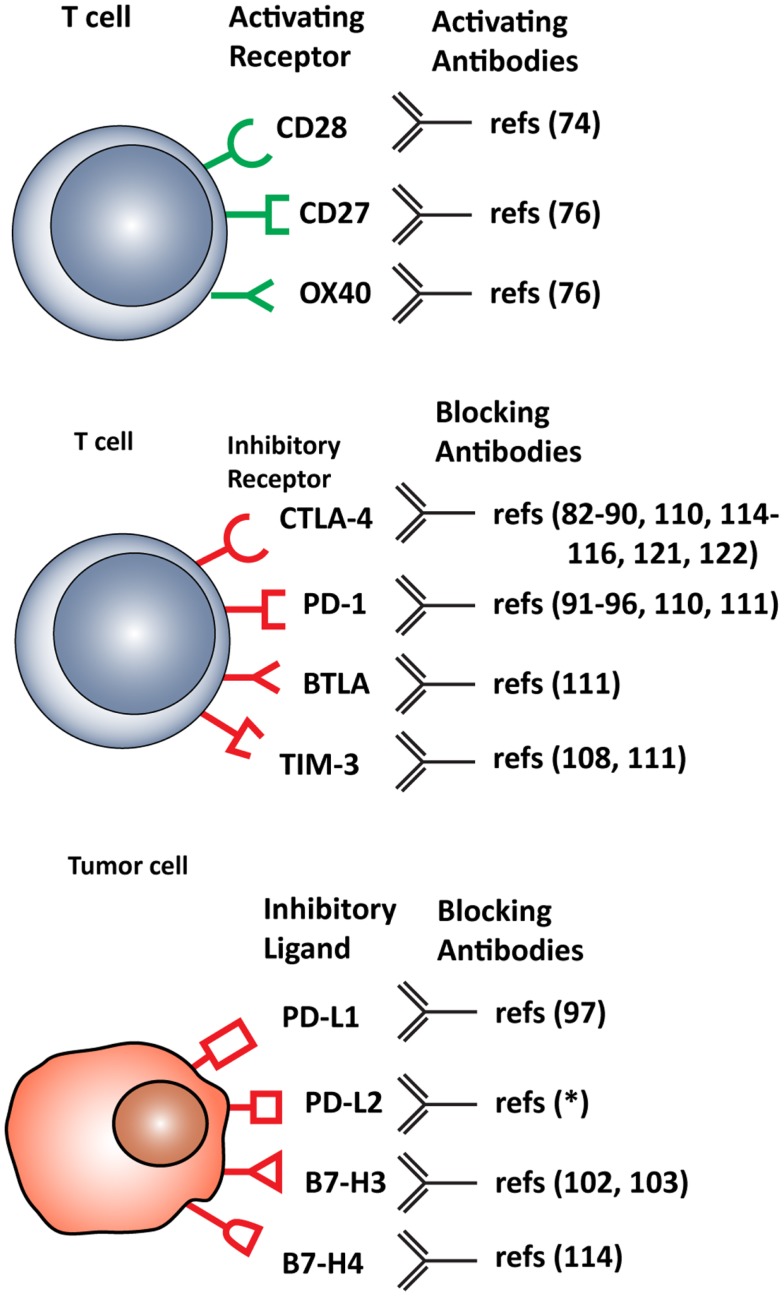
**Stimulatory and inhibitory molecules expressed in the tumor microenvironment targeted for therapeutic intervention**. Schematic visualization of stimulatory and inhibitory receptors expressed on T cells and on various tumor cells. Specific monoclonal antibodies are in development that either function as agonists, enhancing T cell activation, or antagonist, blocking T cell inhibitory molecules. *(ClinicalTrials.gov identifier: NCT00658892).

T cell functions, both priming and effector, can be attenuated by inhibitory checkpoint receptors and ligands expressed by T cells themselves, DCs and other immune cells, or tumor cells. The most important co-inhibitory checkpoint receptors are CTLA-4 and PD-1, in combination with the PD-1 ligands, PD-L1 (B7–H1), and PD-L2 (B7-DC), all belonging to the B7 receptor superfamily. Other B7 family members, such as B7–H3 and B7–H4, and the unrelated receptors herpes virus entry mediator (HVEM), inhibitory receptor Ig-like transcript-3 and -4 (ILT3 and 4), T cell immunoglobulin mucin protein-3 (TIM-3), and lymphocyte activation gene-3 (LAG-3) are also involved in inhibiting T cell function (2) (Figure 2).

#### Cytotoxic T lymphocyte-associated antigen-4

Cytotoxic T lymphocyte-associated antigen-4 is a homolog of the co-stimulatory molecule CD28 and it is exclusively expressed on CD4^+^ and CD8^+^ T cells after activation. Tregs represent an exception, as they constitutively express CTLA-4. In contrast, CD28 is constitutively expressed on all T cell subsets regardless of activation (37–39). CD28 and CTLA-4 are closely related in structure and both bind to the ligands CD80 and 86 present on APCs, such as DCs, macrophages, and B cells (10). Although the expression of CTLA-4 on the cell surface is low compared to CD28, it has a higher affinity for the ligands (37, 40). CTLA-4 receptor ligation leads to inhibition of T cell proliferation, cell cycle progression, and IL-2 synthesis (41, 42). Its cell surface expression is induced by CD28 ligation, implying that it serves as an internal checkpoint, downregulating CD28 stimulation and thereby attenuating immune responses (43). Despite its apparent role in attenuating T cell activation, CTLA-4 seems to be required for effective anti-tumor immunity, as this molecule also affects T cell polarization. *In vivo* studies have shown that CTLA-4-deficiency in mice causes severe lymphoproliferative disorders, promoting a Th2 phenotype (44) while a Th1 phenotype is required for efficient anti-tumor immunity (45, 46).

#### PD-1/PD-L1 and PD-L2

Another inhibitory member of the B7 receptor family is PD-1. This receptor is more widely expressed than CTLA-4, being found on CD4^+^ and CD8^+^ T cells (including Treg cells), B cells, monocytes, and at lower levels on NK cells (47, 48). Its major function is limiting autoimmunity and T cell activity in peripheral tissues in response to infection (49, 50). Tumor cells can exploit these characteristics by inducing expression of PD-1 on tumor-specific T cells, thus suppressing their effector function and eventually leading to T cell exhaustion and immune resistance in the tumor microenvironment (51, 52). Two ligands are known to interact with PD-1: PD-L1 (53) and PD-L2 (54). PD-L1 is expressed on resting and activated T cells, B cells, DCs, mast cells, macrophages, endothelial cells, tumor cells, and other cells within the tumor microenvironment (55–57). This tumor-associated PD-L1 expression was reported to increase apoptosis of infiltrating T cell (52, 58). Interestingly, PD-L1 does not only interact with the PD-1 receptor, but also with CD80 expressed on T cells, inhibiting T cell activation, and cytokine production (59). PD-L2 has a higher affinity for PD-1 than PD-L1, and although its expression was thought to be restricted to APCs, it has been shown to be expressed by normal and cancer-associated fibroblasts, a specific subset of B cells, activated T cells and tumor cells (60). PD-L1 expression on tumor cells is associated with aggressive tumor behavior, poor prognosis, and elevated risk of death, while for PD-L2 such correlations were not significant (60, 61).

#### B7–H3 and B7–H4

Two additional B7 family co-inhibitory ligands are B7–H3 and B7–H4. The receptors for these molecules have not been identified yet, but they are expected to be expressed by activated T cells (62). B7–H3 is constitutively expressed on a wide variety of tissues, and its expression on leukocytes is dependent on inflammatory cytokine stimulation (63). In contrast, expression of B7–H4 is more restricted, being found on T cells, B cells, monocytes, and DCs after activation (64). Many human cancers express B7–H3 and B7–H4, which is generally associated with poor prognosis (65, 66). Furthermore, B7–H3 seems to be upregulated on endothelial cells of the tumor vasculature and on tumor-associated macrophages (TAMs) (67).

#### Other co-inhibitory molecules

Other co-inhibitory receptors, which can be exploited by tumors to dampen anti-tumor immune responses, are HVEM, ILT3 and 4, TIM-3, and LAG-3. HVEM is expressed by immature DCs and interacts with its ligands “B and T lymphocyte attenuator” (BTLA), LIGHT, and CD160, all expressed on T cells (68). HVEM interaction with BTLA inhibits T cell responses, promotes T cell survival, and mediates Treg suppression (2). BTLA and CD160 compete for the same cysteine rich domain of HVEM with a similar affinity, but a different dissociation rate, suggesting a dominant inhibitory role for CD160 (69). It seems that HVEM ligation of BTLA inhibits immune responses against tumor cells, while LIGHT exerts pleiotropic effects to increase this response (70).

Ig-like transcript-3 and -4 are inhibitory receptors both expressed by monocytes, macrophages, and DCs (71, 72). The corresponding ILT3 ligand is not yet known, but since ILT3 can directly suppress T lymphocyte function, it is likely to be expressed on T cells (73, 74). In several cancers, ILT3 has been found to mediate the immune escape mechanism by impairing T cell responses (75). Furthermore, ILT4-expressing DCs block efficient CTL differentiation, a mechanism that is used by tumors, which upregulate ILT4 to evade the immune system (76).

T cell immunoglobulin mucin protein-3 is a checkpoint receptor expressed by IFN-γ-secreting CD4^+^ T helper and CD8^+^ cytotoxic T cells. When interacting with its ligand, galectin-9, it triggers cell death and terminates immune responses driven by these T cells. The most important role of TIM-3 in anti-tumor immunity involves T cell exhaustion and stimulation of MDSC-mediated suppression of T cell responses (77).

Lymphocyte activation gene-3, a CD4 homolog, is an activation-induced cell surface molecule that binds with high affinity to MHC class II on APCs. LAG-3 is expressed by T cells, NK cells, B cells, and plasmacytoid DCs. By binding to its ligand, it inhibits T cell expansion and controls the size of the memory T cell pool (78). When upregulated on Tregs, LAG-3 can modulate suppressive Treg function (79). Furthermore, LAG-3 plays important role in both the homeostatic maintenance and activation-induced expansion of DCs (80). Co-expression of LAG-3 and PD-1 on tumor-infiltrating CD8^+^ T cells, induced by either tumor-derived APCs or cytokines secreted in the tumor microenvironment, contribute to the establishment, and maintenance of an immunosuppressive tumor microenvironment (81).

Taken together, these data show that tumors have evolved intriguing mechanisms to exploit the balance between co-stimulation and co-inhibition by skewing this toward co-inhibition and thus dampening anti-tumor immune responses. In fact, this has become a crucial aspect of immunosuppression in the tumor microenvironment, effective against both natural and induced anti-tumor immunity.

## Clinical Intervention

Strategies to break or neutralize the aforementioned inhibitory mechanisms present in the tumor microenvironment are currently being developed. This can be accomplished by either decreasing activity of suppressive molecules or by increasing activity of stimulatory molecules. Monoclonal antibodies are being produced that bind to co-stimulatory/co-inhibitory receptors and their ligands, and thereby either antagonizing those that suppress immune responses or activating others that amplify immune responses. A number of these are now being tested in the clinic (Figure 2).

### Targeting co-stimulator molecules with agonistic antibodies

As the effector T cells in the tumor microenvironment seem to be immunosuppressed, a logical step would be to develop antibodies that can (re)activate T cell responses in the microenvironment. In this setting, the most attractive target seems to be the co-stimulatory molecule CD28. Agonistic antibodies targeting CD28 were developed and entered clinical testing. However, a trial in which an agonist anti-CD28 monoclonal antibody (TGN1412) was tested has since become a cautionary tale to the power of the immune system. This antibody led to an unexpected release of cytokines (cytokine storm) in the volunteers, causing severe toxicities (82). This incident highlighted the potential dangers of agonistic antibodies and severely decreased the interest in further developing these strategies for many years. Recently, this interest has been re-kindled and a number of agonistic antibodies are being explored. In particular, members of the tumor necrosis factor receptor (TNF-R) family have emerged as targets for enhancing tumor-specific responses. This includes CD27, GITR, 4-1BB, CD30, and OX-40, which are expressed on tumor-specific T cells, and antibodies targeting these molecules are under investigation in several (pre)clinical studies (83). Among these, only anti-4-1BB, and anti-CD30 antibodies had success in clinical trials as mono-therapies. A phase II study with anti-4-1BB treatment showed promising results, but was eventually terminated due to, unexpectedly high, grade 4 hepatitis (84). A recent phase III study of anti-CD30, brentuximab vedotin, as treatment of relapsed patients with Hodgkin lymphoma resulted in a 71% objective response rate (85). CD40, another TNF-R family member, which is expressed on APCs, muscle cells, fibroblasts, and basophils is also being explored as a potential target for immunotherapy (86). Several phase II trials for the treatment of myeloma and diffuse large cell lymphoma are currently testing the efficacy of the humanized anti-CD40 antibody dacetuzumab (10). Also, a new, fully human, anti-CD40 monoclonal antibody was evaluated in a phase I trial and considered safe for further clinical development (87). So, the development of agonistic antibodies is in progress, but the question remains if such indiscriminate activation of T cells will lead to efficient anti-tumor immune responses, or whether the risk of severe adverse effects or autoimmune activation will prove to be too high.

### Targeting co-inhibitory molecules with antagonistic antibodies (blockade of immune checkpoints)

#### CTLA-4 blockade

**Figure 3:**
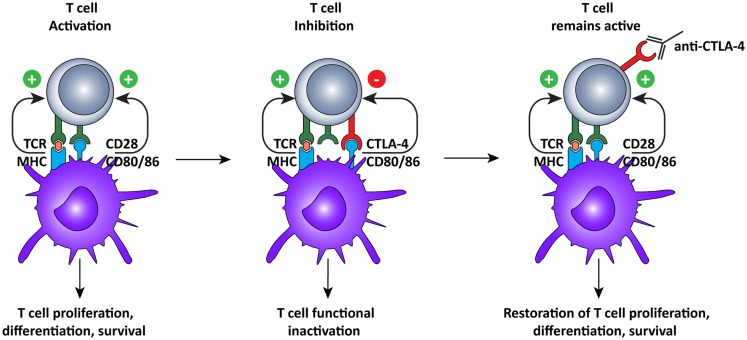
**Cytotoxic T lymphocyte-associated antigen-4 blockade restores T cell activation**. After recognition of MHC:peptide complex by the TCR, the second signal for T cell activation is provided by binding of CD80 or 86 to CD28 on the T cells. This interaction leads to cell surface expression of CTLA-4, which has a higher affinity for CD80/86, thus interrupting the activation signal. Additionally, the signal delivered via CTLA-4 down-regulates T cell function and inhibits excessive expansion of activated T cells. Anti-CTLA-4 monoclonal antibodies bind to CTLA-4, and block the interaction with CD28, which is again free to interact with CD80/86, prolonging T cell activation and amplifying T cell-mediated immunity against tumors.

Just like agonistic antibodies might lead to non-specific activation of the immune system and cause more harm than good, so too was blockade of CTLA-4 questioned initially (Figure 3). Most CTLA-4 expressing T cells are not tumor-specific and *ctla-4* KO mice exhibited a lethal autoimmune and hyperimmune phenotype, predicting immune toxicity in human CTLA-4 blockade (88, 89). However, when CTLA was only partially blocked with antibodies, severe toxicity was prevented and significant anti-tumor responses were observed in mice (90). These pre-clinical results led to the development of two, fully human, anti-CTLA-4 monoclonal antibodies for the treatment of several cancers, including melanoma and renal cell carcinoma (10): ipilimumab, an IgG1 antibody with plasma half-life of 12–14 days (Bristol–Myers Squibb) and tremelimumab, an IgG2 antibody with a plasma half-life of 22 days (Pfizer).

Ipilimumab was tested in a phase II trial but failed to reach its endpoint of tumor regression. Regardless, it was still tested in a large phase III trial and became the first drug to demonstrate survival benefit in patients with advanced melanoma in a randomized trial. Metastatic melanoma patients were treated with ipilimumab, with or without a glycoprotein 100 (gp100) peptide vaccine, or with gp100 alone (91). Patients treated with ipilimumab, with or without gp100, had a 3.5-month survival benefit compared to the group treated with gp100 alone (91). In a second randomized trial, the combination of ipilimumab with standard dacarbazine treatment showed an increase in overall survival of 2.1 months compared to dacarbazine alone (11.2 vs. 9.1 months). Additionally, there was an increase in patients with at least 3 years survival (20.8 vs. 12.2%) (92). In contrast, tremelimumab did not show any significant improvement in survival of patients with metastatic melanoma when tested in a phase III trial in comparison with standard chemotherapy. As a result the development program was abruptly terminated (93).

Although showing promising results, the use of CTLA-4 blockade still presents many challenges for the clinic. There is a significant rate of adverse reaction caused by the treatment, with up to one third of the patients experiencing immune-related serious adverse effects (irSAEs) up to grade 3 or 4, ranging from dermatitis to severe chronic colitis or acute hepatitis (94–96). Furthermore, the efficacy of CTLA-4 blockade as a single treatment seems to be limited to intrinsically immunogenic tumors such as melanoma (97, 98).

#### PD-1 pathway blockade

In contrast to CTLA-4 blockade, PD-1 blockade was expected to be less toxic, based on the different phenotype associated to PD-1 knockout mice. Whereas *ctla-4* KO mice died from a lethal lymphoproliferative disorder at a very young age, some colonies of *pd-1* KO mice lived over a year before expressing lupus-like symptoms (49, 88).

The first fully human anti-PD-1 IgG4 antibody, nivolumab (MDX1106) was tested in a phase I clinical trial. The trial was conducted on patients with different solid tumors and showed promising results, as it was relatively well tolerated (14% grade 3–4 irSAE) and showed anti-tumor activity (99). Long-term follow-up on three patients that participated in the phase I trial (melanoma, renal cell carcinoma, and colorectal cancer) showed the presence of memory T cells that mediated a persistent anti-tumor immune response in the absence of continued therapy, indicating long-term clinical benefit of PD-1 blockade (100). A subsequent, dose-escalating, phase I trial, conducted in melanoma patients, also showed that nivolumab was well tolerated. Immune-related toxicities were mild, less frequent (21% grade 3–4 irSAE), and less severe than those observed with ipilimumab (101–103). This antibody is now being tested as first-line treatment in a phase III trial compared to dacarbazine for treatment of metastatic melanoma (ClinicalTrials.gov identifier: NCT01721772). Two other anti-PD-1 antibodies were tested in clinical trials: lambrolizumab (MK3475) and pidilizumab (CT-011). Lambrolizumab was shown to have a response rate of 38% in patients with melanoma, and induced a durable progression-free survival rate of longer than 7 months and low grade toxic effects (104). Pidilizumab was tested in hematopoietic malignancies, where anti-tumor activity was observed in one patient with follicular lymphoma and one with acute myelogenous leukemia (105). These results seem to indicate that PD-1 blockade, like CTLA-4 blockade, can overcome immunosuppressive mechanisms present in the tumor microenvironment and reactivate pre-existing tumor-specific T cells.

**Figure 4:**
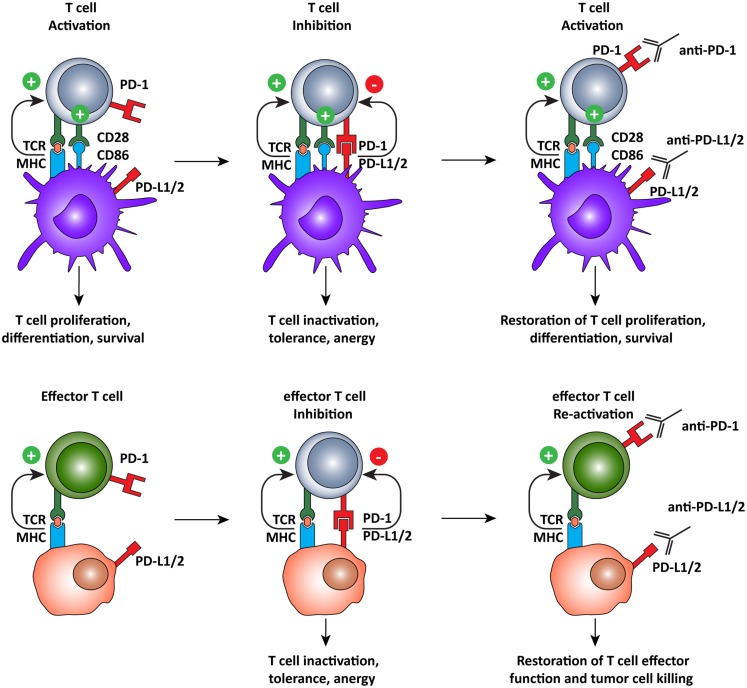
**Programed cell death-1 pathway blockade promotes tumor-specific T cell activation and elimination of tumor cells**. The PD-1 pathway operates on two different levels, regulating both T cell activation by DCs and the effector function of antigen-specific T cells. PD-1 pathway blockade by monoclonal antibodies directed against PD-1, or its ligands, promotes T cell activation by shifting the balance of signals delivered by the DC from suppressive to activating. In the tumor microenvironment, tumor-specific T cells recognize tumor cells but are subsequently inactivated by the expression of PD-L1 or PD-L2 on the tumor cell, inducing tolerance and anergy. When rescued, by blocking the PD-1 pathway, T cells recognize antigen in the periphery and, in the absence of PD-1 engagement, they assume full effector function and eliminate tumor cells.

The ligands of PD-1, PD-L1, and PD-L2, which are expressed on both tumor and normal cells within the tumor microenvironment (55–57, 60), are also interesting targets for immunotherapy (Figure 4). A recent clinical trial of the anti-PD-L1 antibody, BMS-936559, showed durable tumor regression and prolonged stabilization of the disease, with only 9% of patients experiencing grade 3 or 4 irSAE (106). PD-L2 blockade is currently being evaluated in a clinical trial but results are not yet available (ClinicalTrials.gov identifier: NCT00658892). Nonetheless, it appears that targeting PD-L1 and PD-L2 may be a strategy to limit off-target toxicity, while still combating the immunosuppressive tumor microenvironment.

#### B7–H3/H4 blockade

Both B7–H3 and B7–H4 receptors are expressed in tumors of prostate, non-small-cell lung, pancreatic, gastric, and skin cancer (107). In a non-small-cell lung cancer study, high B7–H3 or B7–H4 expression correlated with lymph node metastasis (108). In spite of being expressed on tumor cells, the role of B7–H3 as an inhibitory molecule is still not clear. Some studies have shown that expression of B7–H3 on tumor cells or tumor vasculature is associated with an increased risk of death (109), while others have shown that B7–H3 expression is associated with prolonged patient survival and TIL infiltration (110). In mouse models, B7–H3 overexpression on tumor cells was shown to favor tumor regression (107). However, it has also been reported that antagonistic antibodies could enhance *in vitro* T cell proliferation (111). Altogether, the uncertainty on the exact function of B7–H3 makes its implication in cancer therapy rather difficult. Notwithstanding, a B7–H3 targeting antibody has been developed, which mediates potent cellular toxicity against a broad range of tumor cell types, and is currently being tested in a clinical trial (112). B7–H4 seems to have a clearer role in inhibiting T cell functions (113), and *in vitro* models have shown that antibody-mediated blockade of B7–H4 could restore anti-tumor T cell responses, making it an interesting target for clinical application (114).

#### Blockade of other immune checkpoints

Up till now, clinical intervention strategies have focused primarily on the B7 family, as highlighted above; other immune checkpoint pathways are not as well established and therefore research has been limited to pre-clinical, *in vitro* studies or mouse models. Nonetheless, these may prove to be important therapeutic targets in the future. The interaction of HVEM with several ligands, such as BTLA, CD160, and LIGHT, makes the balance between co-stimulatory and co-inhibitory signals rather complex. It also seems that signaling is bidirectional, depending on the specific combination of interactions. Therefore, immune checkpoint blockade in this pathway is not as straightforward as with other molecules (68, 96). Further delineation of the complex HVEM/BTLA/CD160/LIGHT pathway is required to elucidate the possibilities in immune blockade therapies.

The inhibitory receptors ILT3 and 4 also play an important role in the regulation of the immune response. In patients with melanoma, and carcinomas of the colon, rectum, and pancreas, ILT3 was reported to mediate immune escape mechanism, resulting in largely unsuccessful immune therapies (75). Soluble ILT3 protein induces differentiation of CD8^+^ T cell and impairs T cell responses (75, 115). This could be restored by anti-ILT3 antibody or depletion of the soluble ILT3 from the serum. Thus, blocking ILT3 may prove to be an important adjuvant in immunotherapy. ITL4 upregulation on DCs was reported to cause blockade of cytotoxic T cell differentiation (76). Blockade of this receptor would therefore also be useful to augment DC function and enhance immune responses to cancer.

On the other hand, blockade of TIM-3 seems more feasible, as anti-TIM-3 displayed modest prophylactic and therapeutic activity against a small fraction of sarcomas in a mouse model. Furthermore, IFN-γ production from CD8^+^ cells, but not from CD4^+^ cells, was shown to be critical for the anti-tumor effect of the anti-TIM-3 treatment (116). TIM-3 blockade seems to mainly stimulate anti-tumor responses via NK cell-dependent mechanisms, while blockade of another family member, TIM-4, induces CD8^+^ cytotoxic T cells (117).

## Combinatorial Immunotherapies

Up till now, immune checkpoint blockade has mostly been developed as monotherapy with marginal efficacy, but the use of these immune checkpoint blockades in combinatorial regimens might improve clinical efficacy. Although these therapies could be combined with the usual suspects, radio- and chemo-therapy, the most benefit might reside in the combination with other immunotherapeutic approaches. However, extra care is warranted, as manipulation of the tightly controlled balance of immune activation vs. inhibition could be dangerous.

### Combing immune checkpoint blockades

As CTLA-4 blockade and PD-1 pathway blockade target different mechanisms of T cell inactivation, there is a rational for expecting synergy when combining both these immune checkpoint blockades. Taking the high prevalence of irSAEs associated with these treatments when used as monotherapy into account, combining them is a risky proposition at best. Nevertheless, this combination treatment (anti-CTLA-4 mAb, ipilimumab and anti-PD-1 mAb, nivolumab) was tested in a recent, dose-escalating, phase I trial, and the results were very promising. The highest dose showed a 53% objective response and all patients had at least 80% tumor shrinkage. As might be expected, immune toxicity was higher than with monotherapy but this was a small increase compared to the increase in clinical response (118). Although the patient numbers in this trial were small, there was clear synergistic effect when combining these two immune checkpoint blockades. This is currently being confirmed in a phase III trial.

Programed cell death-1 pathway blockade in combination with other co-inhibitory molecules has also proven to be potentially useful. Blockade of the HVEM ligand, BTLA, in combination with PD-1 and TIM-3 blockades enhanced IL-2-producing CD8^+^ T cell expansion in an *in vitro* melanoma model (119). Also, when anti-PD-1 and anti-TIM-3 antibodies are combined, a significant decrease of tumor size was found, compared to PD-1 blockade alone (99). Since LAG-3 and PD-1 are co-expressed on CD4^+^ and CD8^+^ T cells, several combinatorial therapies have been explored. Frequency and effector function of CD8^+^ T cells were increased after LAG-3 and PD-1 blockade in a mouse model of epithelial ovarian cancer (81). Additionally, another *in vivo* study, applying a dual anti-LAG-3/anti-PD-1 antibody therapy showed a markedly improvement of the overall condition of mice challenged with tumor, that were resistant to single antibody treatment (120).

### Combining CTLA-4 blockade with DC vaccination

The main problems encountered with anti-CTLA-4 treatment are the resistance of advanced tumors, due to a strong tumor-induced T cell tolerance, which may be partially PD-1 pathway mediated, and a lack of tumor specificity (121). Thus, a novel and potentially successful strategy would be the combination of DC vaccination with CTLA-4 blockade. This is supported by several pre-clinical tumor models, showing that CTLA-4 blockade on its own is not very potent in triggering a specific anti-tumor response, but when combined with agents that prime immune responses, such as DC vaccination, it might become very effective. In a study using a EL4 lymphoma mouse model, the administration of a single dose DC vaccination in combination with anti-CTLA-4 monoclonal antibody resulted in the rejection or retarded tumor growth in 60% of the challenged tumor mice, while either the vaccine or CTLA-4 blockade were ineffective when administered alone (122). The combination of CTLA-4 blockade and vaccination with B16 or SM1 cells, genetically modified to express GM-CSF, showed enhanced efficacy and tumor regression when administered in a B16 melanoma model and SM1 mammary carcinoma model, respectively. In the same experimental set up, monotherapy was again ineffective (123, 124). Taken together, these data suggest that CTLA-4 blockade in combination with DC vaccination could break tolerance to tumor-specific antigens, resulting in tumor clearance, and long-term host immunity after tumor re-challenge.

### Combining PD-1 pathway blockade with DC vaccination

In parallel with therapies which combine CTLA-4 blockade with DC vaccination, strategies for interfering with PD-1 pathway to enhance DC vaccination are being explored in pre-clinical studies. Administration of poly(I:C), a TLR3 agonist, as a tumor vaccine adjuvant was shown to selectively upregulate PD-L1 expression on mouse CD8α^+^ DCs. Although the CD8α^+^ DCs were able to promote cross-priming of CD8^+^ T cells, there was a lack of expansion of the primed tumor antigen-specific CD8^+^ T cells. This resulted in a failure to establish an anti-tumor immune response, suggesting that TLR3-induced PD-L1 expression on DCs may act as a negative regulator of CD8^+^ T cells expansion. Thus, blockade of PD-L1 on poly(I:C)-activated DCs might improve the anti-tumor efficacy of DC-based vaccines (125). In fact, in a B16 murine melanoma model treated with tumor peptide-pulsed DCs, concurrent systemic administration of anti-PD-L1 antibody resulted in a higher number of melanoma peptide-specific CD8^+^ T cells. Surprisingly, in spite of the increased number of tumor-specific T cells, there was no significant reduction in tumor growth (126). Additionally, blockade of PD-1/PD-L1 immune checkpoint in a murine breast cancer model was shown to effectively augment DC function in the stimulation of tumor-specific T cell mediated cytotoxicity, leading to efficient induce anti-tumor immunity (127). Together, these studies support blocking of the PD-1 pathway as a means to enhance the efficacy of DC vaccination.

### Combining CTLA-4 blockade with other cancer treatments

Combining CTLA-4 blockade with other immunotherapeutic approaches or targeted therapies is also proven to be beneficial in several mouse models and has also entered clinical trials. The combination of the GM-CSF-engineered allogeneic vaccine GVAX with ipilimumab showed an improved overall survival of 29.2 months in patients with metastatic castration-resistant prostate cancer, but also displayed increased toxic effects when compared to therapy with either agent alone (128). In a recent phase I trial, ipilimumab was combined with the BRAF inhibitor vemurafenib in melanoma patients with the V600E BRAF mutation. However, the study was closed due to unforeseen hepatotoxicity, again highlighting the need for extreme care when combining these treatment modalities (129). In a long-term study, patients with metastatic melanoma treated with ipilimumab and IL-2 showed a 17% complete response rate, which is promising but still needs to be verified in a randomized trial (130).

## Discussion and Future Perspective

Although cancer immunotherapy development is now flourishing and recognized as a novel important treatment modality by oncologists, it had a rough start, as most immunotherapeutic agents were not effective in early trials (131). Over the years, the field of immunotherapy has evolved and matured. Growing knowledge about the immunosuppressive tumor microenvironment has provided some new promising checkpoint targets, as described above. This has all resulted in FDA-approved treatment modalities such as ipilimumab and Sipuleucel-T. Notably, the introduction of ipilimumab to the clinic has provided a boost to cancer immunotherapy, particularly keeping in mind that ipilimumab is the first anti-cancer treatment approved that does not target the tumor but rather targets the immune system. However, despite having clear therapeutic benefits and showing the possibility of long-term survival, there are still some challenges ahead. The first problem is the observed spectrum of toxicity or irSAEs, causing inflammatory and autoimmune reactions. This was to be expected on the basis of the pre-clinical mouse models, but is nonetheless a serious problem. In clinical trials, up to 25–30% of patients treated with ipilimumab suffer from grade 3 to 4 SAEs, including dermatitis, colitis, and hypophysitis (94). Unfortunately, there is no correlation between anti-tumor effect and the severity of these side effects, meaning that the patients experiencing these irSAE do not necessarily benefit from an anti-tumor effect. In this regard, blockade of PD-1 or PD-L1 has proven to be a much milder treatment alternative. In theory, blockade of CTLA-4 seems to be more effective than PD-1 pathway blockade, as it might lead to the activation or induction of new tumor-specific T cells, in addition to (re)activation of pre-existing tumor-specific T cells. However, both CTLA-4 and PD-1 pathway blockade seem to have similar clinical efficacy, but PD-1 pathway blockade is reported to have significantly fewer instances of irSAE.

A second drawback of immune checkpoint blockade is the lack of specificity. These treatment modalities are designed to “release the brakes” on the immune system, leading to indiscriminate immune activation, which is the cause of the irSAEs. This also means that only patients that already have pre-existing, naturally induced, tumor-specific T cells, which are being suppressed by these immune checkpoints, will benefit. Although CTLA-4 blockade is thought to be able to activate new tumor-specific T cells, this has never been proven in humans, and up till now this therapy seems to be the most effective in immunogenic tumors. Furthermore, a recent study has shown that patients whose tumors had higher expression of genes involved in immune function before the start of the treatment responded better to ipilimumab. Furthermore, expression of genes associated with T cell responses were increased after ipilimumab therapy. These findings support the concept that ipilimumab may be more efficacious in subjects who have pre-existing natural, albeit ineffective, anti-tumor immune responses (97).

**Figure 5:**
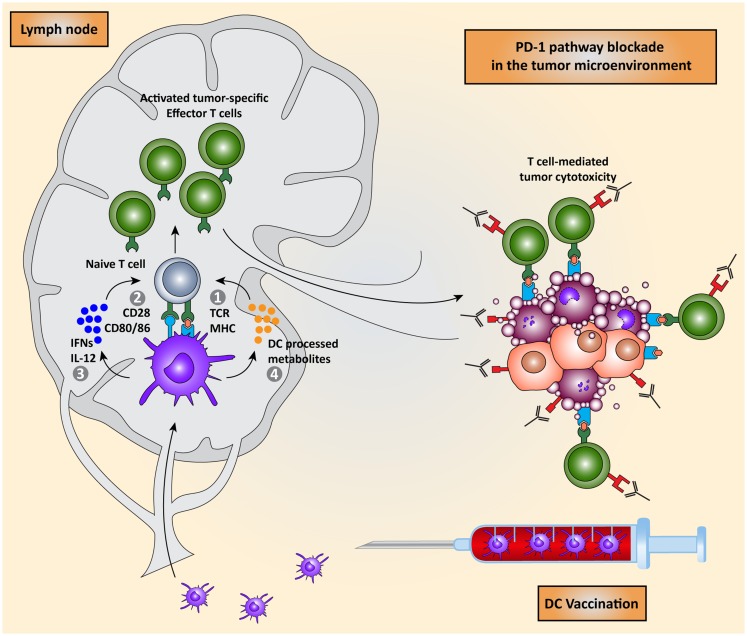
**Combining DC vaccination with immune checkpoint blockade**. DC vaccination of cancer patients leads to the induction of tumor-specific T cells that migrate to the tumor microenvironment. PD-1 pathway blockade synergistically potentiates the effects of DC vaccination by blocking PD-1/PD-L1 induced immunosuppression leading to enhanced tumor cell killing.

Combining non-toxic DC vaccination with immune checkpoint blockade might be a good combination, exploiting the advantage of DC vaccination: the induction of tumor-specific T cells to compensate for the lack of specificity in checkpoint blockade. Conversely, this combination might also compensate for the lack of potency of the DC-induced tumor-specific T cells, by blocking the expression of inhibitory molecules in the tumor microenvironment (Figure 5). A recent phase II trial, assessing safety and dosage, showed that the combination of DC vaccination with dose escalation of the CTLA-4 blocking antibody, tremelimumab, resulted in objective and durable tumor regressions, while irSAE were limited to grade 3 (132, 133). This indicates that this combination regiment in practice does not lead to extra toxicity compared to CTLA-4 blockade and might be even less toxic. Although not directly compared, or in combination with DC vaccination, recent results in clinical trials indicate that PD-1 pathway blockade are more active and less toxic than CTLA-4 blockade. This might be due to the more tumor-specific mode of immune activation. Additionally, PD-1 blockade might also provide the possibility of using biomarkers to select patients that will respond. In the nivolumab trial, 9 out of 25 patients with PD-L1 expression in the tumor responded to treatment while none of the 17 patients whose tumor did not express PD-L1 responded. Additionally, a recent study identified increased PD-1 expression on tumor-specific CD8^+^ T cells in melanoma patients, indicating that the PD-1 pathway is actively contributing to suppressing immune response in melanoma patients. Together, these results warrant for a phase I/II trial combining DC vaccination with PD-1 pathway blockade where patients are selected for increased PD-1 expression on CD8^+^ T cells or expression of PD-L1 by their tumor (103, 134).

Finally, it should be mentioned that also other options exist to combat inhibitory molecule expression within the tumor microenvironment. Recent studies have indicated that chemotherapeutic drugs can potentiate the immune system via the so-designated “off-target effects” (135). For example, platinum-based chemotherapeutics were shown to downregulate PD-L1 on DCs while also downregulating PD-L2 on both DCs and tumor cells. This resulted in enhanced T cell activation and increased tumor cell recognition (136, 137). Chemotherapy may therefore also potentiate the effect of immunotherapy by improving DC maturation and function and eliminating suppressive cells (138).

In summary, cell-based immunotherapeutic approaches, such as DC vaccination, are promising strategies for cancer treatment. After years of optimization, these therapies are succeeding in inducing tumor-specific T cells in cancer patients. Unfortunately, so far this was insufficient to produce clear clinical benefits, albeit long-lasting responses were seen in a small proportion of the patients. A major factor hampering these novel therapies is the immunosuppressive tumor microenvironment. When migrating to the tumor site, tumor-specific T cells end up in an environment specialized in suppressing anti-tumor immune responses. Tumor cells accomplish this in large part by exploiting immune checkpoints, designed to dampen immune responses after infection and prevent autoimmunity. Recent antibody-based immunotherapeutic approaches, specifically designed to block these T cell inhibitory pathways, facilitate effector T cells to attack the tumor. The main drawback of checkpoint blockade antibodies is their lack of specificity, especially since it is not possible to determine in advance if tumor-specific T cells are present.

In this review, we highlighted the crucial role of the intricate regulatory molecular networks governing T cell activation and effector function, immune checkpoints, in the context of anti-tumor immunity and how these mechanisms are hijacked by tumors in order to suppress immune responses. More importantly, we discussed the use of immune checkpoint blockades as cancer treatment and provided a rationale for combining these with DC vaccination as a potentially superior alternative to blocking multiple immune checkpoints. Altogether, our growing knowledge about the immunosuppressive tumor microenvironment, and especially how it can be manipulated in a therapeutic setting, has opened up a fantastic opportunity to synergistically combine checkpoint blockade, especially PD-1 pathway blockade, with DC vaccination or adoptive T cell transfer. This will result in a powerful combination regiment leading to tumor clearance and immunological memory, which can mediate long-lasting tumor regression.

## Conflict of Interest Statement

The authors declare that the research was conducted in the absence of any commercial or financial relationships that could be construed as a potential conflict of interest.
